# Machine-learning-based COVID-19 mortality prediction model and identification of patients at low and high risk of dying

**DOI:** 10.1186/s13054-021-03749-5

**Published:** 2021-09-08

**Authors:** Mohammad M. Banoei, Roshan Dinparastisaleh, Ali Vaeli Zadeh, Mehdi Mirsaeidi

**Affiliations:** 1 Department of Critical Care Medicine, University of Calgary, Alberta, Canada; 2 Department of Biological Science, University of Calgary, Alberta, Canada; 3 Division of Pulmonary and Critical Care Medicine, Johns Hopkins University, Baltimore, MD 21218 USA; 4 Division of Pulmonary and Critical Care, Miami VA Medical Center, Miami, FL USA; 5 Division of Pulmonary and Critical Care, Department of Medicine, University of Miami, Miami, FL USA

**Keywords:** COVID-19, Prediction model, Machine learning, SARS-CoV-2, Mortality

## Abstract

**Background:**

The coronavirus disease 2019 (COVID-19) pandemic caused by the SARS-Cov2 virus has become the greatest health and controversial issue for worldwide nations. It is associated with different clinical manifestations and a high mortality rate. Predicting mortality and identifying outcome predictors are crucial for COVID patients who are critically ill. Multivariate and machine learning methods may be used for developing prediction models and reduce the complexity of clinical phenotypes.

**Methods:**

Multivariate predictive analysis was applied to 108 out of 250 clinical features, comorbidities, and blood markers captured at the admission time from a hospitalized cohort of patients (*N* = 250) with COVID-19. Inspired modification of partial least square (SIMPLS)-based model was developed to predict hospital mortality. Prediction accuracy was randomly assigned to training and validation sets. Predictive partition analysis was performed to obtain cutting value for either continuous or categorical variables. Latent class analysis (LCA) was carried to cluster the patients with COVID-19 to identify low- and high-risk patients. Principal component analysis and LCA were used to find a subgroup of survivors that tends to die.

**Results:**

SIMPLS-based model was able to predict hospital mortality in patients with COVID-19 with moderate predictive power (*Q*^2^ = 0.24) and high accuracy (AUC > 0.85) through separating non-survivors from survivors developed using training and validation sets. This model was obtained by the 18 clinical and comorbidities predictors and 3 blood biochemical markers. Coronary artery disease, diabetes, Altered Mental Status, age > 65, and dementia were the topmost differentiating mortality predictors. CRP, prothrombin, and lactate were the most differentiating biochemical markers in the mortality prediction model. Clustering analysis identified high- and low-risk patients among COVID-19 survivors.

**Conclusions:**

An accurate COVID-19 mortality prediction model among hospitalized patients based on the clinical features and comorbidities may play a beneficial role in the clinical setting to better management of patients with COVID-19. The current study revealed the application of machine-learning-based approaches to predict hospital mortality in patients with COVID-19 and identification of most important predictors from clinical, comorbidities and blood biochemical variables as well as recognizing high- and low-risk COVID-19 survivors.

## Background

The COVID-19 disease has resulted in a substantial cause of morbidity and mortality across the world [1]. COVID-19 disease presents with a wide range of clinical features spanning from no symptoms to multi-organ failure [2]. Although SARS-CoV-2 mainly affects the lungs and is associated with developed acute respiratory distress syndrome (ARDS), it can impact cardiovascular, neurological, renal, and vascular complications associated with high mortality [3]. The precise prognostication of COVID-19 clinical outcome is more challenging due to the high variability in disease severity that could essentially be helpful for effective triage and efficient allocation of limited resources (i.e., beds, ventilators). More accurate subclassification of COVID-19 is essential for prognostication and identification of severity [4].

It has been shown that the pathological, physiological, and immunological responses do not sufficiently discriminate patients with non-severe and severe form due to the high level of complexity of these features [4]. A combination of clinical features and biochemical markers has been studied to identify the clinical subtype of COVID-19. Data mining and machine learning (ML) approach could potentially be applied to such diverse multimodal data for the classification of patients with COVID-19 [4]. Therefore, AI has been used for the diagnosis of COVID-19 pneumonia, stratification of patients and developing a prediction model of patterns of spread [5]. AI- and ML-based approach can be used as either diagnostic tool or a prognostic model to predict outcome [6]. Many studies have characterized the association of major risk factors with the COVID mortality such as higher age, cardiovascular disease, chronic respiratory disease, diabetes, hypertension, smoking history, and obesity [7]. However, they could not be strong individual predictors mainly through using conventional statistical analysis due to high degree of complexity and collinearity among the data.

In the present study, we aimed to apply ML-based algorithms to generate a mortality prediction model for hospitalized COVID-19 patients as well as classification of patients to verify the low- and high-risk groups.

## Methods and materials

### Data collection

In a retrospective study, we used clinical data from 400 patients with a polymerase chain reaction (PCR) test confirmed patients with COVID-19. Data were collected from patients admitted at the University of Miami Hospital, Miller School of Medicine, Miami, FL, USA, since June 2020. A total of 250 variables including biochemical and clinical data were collected at various times (hospital admission, ICU admission, hospital discharge). The admission time data were considered as the data at presentation. These data including demographic variables in addition to comorbidities, patients’ vitals, anthropometric measurements, chronic treatments, and laboratory works were obtained from the patient’s electronic records. In the processing dataset, the missing values level of each variable were found among the current cohort. The maximum level of missing values was 7% among the variables. Using imputation methods, new data were created by replacing all missing values with the estimated values using mean imputation. Continuous variables were median fold normalized, log-transformed, and univariance scaled before statistical analysis.

### Definitions of variables

Table 1 summarizes patients’ demographics, clinical variables, comorbidities, and their association with hospital mortality and survival of patients with COVID-19.

**Table 1:** Distribution of patients’ demographics, clinical variables, and comorbidities between hospital mortality and survival of patients with COVID-19

	Variables	Hospitalized death		*P* value
		Yes	No	
1	Male	22 (70.9)	118 (53.8)	0.085
2	Age (years) M ± SD	78.1 ± 10.6	60.58 ± 16.78	< 0.0001*
3	Height (cm) M ± SD	161.19 ± 31.69	167.04 ± 15.89	0.106
4	Weight (lb.) M ± SD	176.93 ± 25.77	180.37 ± 47.41	0.467
5	GCS M ± SD	13.50 ± 3.89	14.80 ± 1.50	0.009*
6	Temperature M ± SD	100.04 ± 1.41	99.31 ± 6.37	0.528
7	Respiratory rate (RR)	27.68 ± 15.23	22.12 ± 6.2	< 0.0001*
8	Heart rate M ± SD	95.52 ± 25.44	93.34 ± 20.91	0.599
9	Blood pressure (systolic) M ± SD	123.58 ± 30.77	130.54 ± 24.15	0.149
10	Blood pressure (diastolic) M ± SD	67.58 ± 19.64	74.63 ± 16.68	0.032
11	O_2_ saturation M ± SD	93.63 ± 5.81	93.35 ± 5.57	0.118
12	ynO_2_ M ± SD	0.76 ± 0.43	0.56 ± 0.49	0.058
13	FiO_2_% M ± SD	81.29 ± 29.71	50.22 ± 59.81	0.042*
14	O_2_ flow (lpm) M ± SD	23.07 ± 21.79	8.55 ± 14.0	0.002*
15	Nursing home	12 (38.7)	26 (11.9)	0.001*
16	H1N1	21 (67.7)	135 (61.6)	0.295
17	European American	21 (67.7)	126 (57.5)	0.332
18	Hispanic	153 (67.4)	86 (58.1)	0.068
19	African-American	4 (12.9)	57 (26.02)	0.074
20	Shelter/homeless	0	7 (3.19)	0.602
21	Asian	1 (3.2)	3 (1.36)	0.413
22	> one race	2 (6.4)	18 (8.21)	1.00
23	Patient delay ≥ 7	6 (19.35)	54 (24.65)	0.788
24	Smoking	1 (3.2)	16 (7.3)	0.702
25	Alcohol	4 (12.9)	62 (28.31)	0.166
26	Flu vaccine	4 (12.9)	46 (21)	1.00
27	Pneumonia vaccine	5 (16.12)	41 (18.72)	0.491
28	Cough	19 (61.2)	126 (57.5)	0.300
29	Sore throat	2 (6.4)	6 (2.73)	0.207
30	Rhinorrhea	1 (3.2)	9 (4.10)	1.000
31	Sputum	2 (6.4)	21 (9.58)	1.000
32	Chest pain	0	34 (15.52)	0.031*
33	Dyspnea	24 (77.41)	132 (60.27)	0.067
34	Hemoptysis	0	4 (1.82)	1.000
35	Fever	19 (61.2)	136 (62.1)	0.063
36	Chills	6 (19.35)	66 (30.13)	0.500
37	Headache	0	21 (9.58)	0.140
38	Myalgia	6 (19.35)	54 (24.65)	0.816
39	Abdominal pain	2 (6.4)	31 (14.15)	0.545
40	Diarrhea	4 (12.9)	40 (18.26)	0.793
41	Nausea–vomiting	4 (12.9)	34 (15.52)	1.000
42	Altered Mental Status (AMS)	10 (32.25)	19 (8.67)	< 0.0001*
43	Anosmia (loss of smell)	0	6 (2.73)	1.000
44	Ageusia (loss of taste)	0	3 (1.36)	1.000
45	Chronic treatment	23 (74.19)	124 (56.62)	0.072
46	On any chemotherapy	3 (9.67)	15 (6.84)	0.464
47	Home O_2_	5 (16.12)	6 (2.73)	0.006*
48	Inhaled steroid	3 (9.67)	23 (10.50)	1.000
49	Prednisone	3 (9.67)	14 (6.39)	0.442
50	ACE inhibitors	9 (29.03)	32 (14.61)	0.061
51	ARBs	6 (19.35)	27 (12.32)	0.253
52	Statins	12 (38.7)	65 (29.6)	0.290
53	Prior ER visit (on past 12 months)	10 (32.25)	81 (36.98)	1.000
54	Any prior hospitalization	12 (38.7)	83 (37.89)	0.378
55	Consolidation on the imaging	13 (41.93)	34 (15.52)	0.002*
56	Pleural effusion on the imaging	6 (19.35)	25 (11.41)	0.250
57	Pulmonary infiltrates on the imaging	17 (54.83)	103 (47.03)	0.568
58	Asthma	2 (6.4)	28 (12.78)	0.548
59	Pulmonary embolism (PE)	0	8 (3.65)	0.601
60	COPD	4 (12.9)	16 (7.30)	0.273
61	Emphysema	1 (3.2)	4 (1.82)	0.486
62	Bronchiectasis	0	2 (0.91)	1.000
63	CHF	6 (19.35)	8 (3.65)	0.003*
64	CAD	11 (35.48)	14 (6.39)	< 0.0001*
65	AMI	6 (19.35)	3 (1.36)	< 0.0001*
66	AFib	3 (9.67)	19 (8.67)	0.740
67	Hypertension	26 (83.87)	126 (57.53)	0.002*
68	Peripheral vascular diseases	2 (6.4)	12 (5.47)	0.676
69	Stroke	4 (12.9)	14 (6.39)	0.138
70	Dementia	8 (25.08)	17 (7.76)	0.004*
71	Chronic renal failure (CRF)	6 (19.35)	21 (9.58)	0.107
72	Hemodialysis	3 (9.67)	6 (2.73)	0.079
73	Liver diseases	0	8 (3.65)	0.601
74	Diabetes	21 (67.7)	59 (26.94)	< 0.0001*
75	Peptic ulcer disease (PUD)	1 (3.2)	11 (5.02)	1.000
76	Leukemia	1 (3.2)	4 (1.82)	0.473
77	Lymphoma	1 (3.2)	7 (3.19)	1.000

In this table, the patient’s level of consciousness, when it was available, is shown based on Glasgow Coma Scale (GCS). We mentioned the patient’s temperature in Fahrenheit. Respiratory rate (RR) indicates the number of breaths per minute, and the heart rate (HR) demonstrates the number of heart beats per minute. The patients’ systolic and diastolic blood pressure (BP) is presented in millimeters of mercury. The percentage of oxygen-saturated hemoglobin to the total hemoglobin is displayed by O_2_ saturation, and ynO_2_ shows whether the patient was on oxygen during the hospitalization. The percentage of the oxygen that the patient inhales is presented by FiO_2_ (the fraction of inspired oxygen). O_2_ flow (lpm) indicates the required oxygen flow in liters per minute. Nursing home shows whether the patient was in a nursing home or long-term care facility before hospitalization. Patient delay ≥ 7 is used to define patients who delayed at least seven days to seek medical assistance after the onset of symptoms.

Smoking and alcohol are used to show the patient’s history of exposure to these toxins. The patient’s vaccination status against influenza (flu vaccine) and pneumonia (pneumonia vaccine) is included as per medical records or informed by the patient at the time of inclusion in the study.

Altered Mental Status (AMS) refers to any decline in the patient’s mental capacity noted through the physical exam. The loss of sense of smell and taste is displayed as anosmia and ageusia. We collected data related to the use of any chronic treatments or chemotherapy. Home O2 shows whether the patient was on supplemental oxygen therapy at home. We have also determined whether the patients are on local (inhaled steroids) or systemic corticosteroids (prednisone). ACE inhibitors indicate that the patient was on chronic treatment with angiotensin-converting enzyme inhibitors, and ARBs refer to the chronic use of the angiotensin ll receptor blockers. To evaluate the predictive value of imaging tests, we have collected data about radiological findings in the patient’s chest X-ray. Consolidation on the imaging refers to the existence of dense material in the alveoli and small airways. The presence of excess fluid accumulation in pleural space is listed as pleural effusion on the imaging, and the existence of dense material in the interstitium is mentioned as pulmonary infiltrates on the imaging.

The chronic health conditions of participants were collected to determine the impact of comorbidities on the outcome. These conditions include diabetes, chronic obstructive pulmonary disease (COPD), emphysema, pulmonary embolism (PE), bronchiectasis, interstitial lung disease (ILD), congestive heart failure (CHF), coronary artery disease (CAD), acute myocardial infarction (AMI), atrial fibrillation (AFib), hypertension, peripheral vascular disease, stroke, dementia, any stage of chronic renal failure (CRF), liver disease, peptic ulcer disease (PUD), connective tissue disorder, leukemia, lymphoma, dependence on hemodialysis, and asthma.

### Statistical analysis

To establish a prediction model, we used the statistically inspired modification of partial least square (SIMPLS) analysis for the clinical data and blood markers collected at admission time. SIMPLS, an algorithm of PLS (a linear machine learning method) [8, 9], was carried out with two training and validation sets. To develop the best prediction model, SIMPLS-based prediction model was built using all variables as primary model. SIMPLS predicts the outcome response to variables by fitting a regression model (*Y* = *XB*) that is derived using the variables. Since all variables were not important to predict outcome, secondly variable reduction in SIMPLS was done to characterize useful predictor in explaining variation in the predictor variable as well as their correlation to outcome. Variable reduction was applied to remove out the factors that were not useful in predicting outcome according to the variable important for the projection (VIP) value of each variable. VIP values were obtained through weighted sum of squares of the weights using SIMPLS analysis [10]. Thus, the contribution of variables in the SIMPLS models was assessed using VIP score. Based on the general agreement, the variables with the VIP values more than 1.0 were considered as important predictors [11]. The variables with lack of predictive ability (VIP < 1.0) were removed from the basic prediction model.

The prediction model was created using the most differentiating clinical and biochemical variables (VIP > 1.0). The validation set automatically and randomly was created including 35% of out 250 hospitalized patients. In the absence of external validation cohort, splitting study cohort into training and validation sets is most known approach for internal validation of multivariate and machine-learning-based prediction mode.

SIMPLS was performed using the leave-one-out method of cross-validation (CV). The CV method is also known as internal validation. SIMPLS analysis was assessed using *Q*^2^, the goodness for predictability, and *R*^2^*Y*, the goodness of variability. The best model was selected based on the number of factors for which *Q*^2^ was larger and had not started decreasing with the highest *R*^2^*Y*. The range of *R*^2^ and *Q*^2^ varies between 0 and 1, the higher level showing higher predictive accuracy. Depending on data, the thresholds for the model performance change, generally *R*^2^ greater than 0.67 and 0.33, are considered as high and moderate predictive accuracy, respectively. Although *Q*^2^ value greater than zero shows the model is predictive, *Q*^2^ value with a range 0.2–0.4 is considered as a model with moderate predictability. Close *R*^2^ and *Q*^2^ show a lack of overfitting and the SIMPLS model works independently of the specific data [12, 13].

The *Q*^2^ and *R*^2^*Y* were computed using the training set and were verified using the validation set that make the model more realistic. Validation set was randomly selected from study cohort in a blinded approach.

Also, the partition analysis was used to creating a decision tree of the partition of data according to a relationship between the outcome and predictors. The data were partitioned into training and validation sets. The partition algorithm was to search all possible splits of predictors to best predict the response. The most differentiating clinical predictors obtained by SIMPLS were used for the partition analysis. AUC were obtained for both training and validation sets through the partition analysis based on the most important variables that were selected strong predictors in the SIMPLS-based prediction model.

We also used the partition analysis to obtain cutting value for either continuous or categorical (nominal or ordinal) variables such as age, heart rate, respiratory rate, and BMI. PCA and clustering were performed to identify subgroups particularly survivor subgroups. PCA was carried out in two steps. The first step was based on all variables to find outliers and trends and the step was using the most differentiating predictors obtained by SIMPLS. PCA and clustering were to help to find a subgroup of survivors that tends to hospital death. Latent class analysis (LCA) was carried to cluster the patients with COVID-19. Clustering was to help to identify the high-risk patients for dying. All paraclinical variables were normalized and transformed to use independently or in combination with clinical data for predicting hospital mortality.

## Results

### Patients’ characteristics

A total of 250 hospitalized patients with RT-PCR confirmed COVID-19 enrolled in the study, and 31 (12.4%) patients died in hospital. Table 1 shows the demographic characteristics, comorbidities, and outcomes of patients with COVID-19 that were admitted to MICU. The table shows, age, respiratory rate, FiO_2_%_,_ O_2_ flow (lpm), having been in nursing home, chest pain, Altered Mental Status (AMS), having been on home supplemental O_2_ therapy, pulmonary consolidation on the imaging, chronic heart failure (CHF), coronary artery disease (CAD), acute myocardial infarction (AMI), dementia, hypertension, and diabetes mellitus were significantly different between the two cohorts. Table 2 shows the laboratory variables among survived and died patients.

**Table 2:** Distribution of patients’ laboratory variables between hospital mortality and survival of patients with COVID-19

Variables	Hospitalized death		*P* value	Normal range
	Yes	No		
Leukocytes (10^3^/µL)	11.75 ± 7.69	7.73 ± 4.55	< 0.0001*	4.5–11
Neutrophils (10^3^/µL)	13.34 ± 14.92	8.59 ± 13.59	0.074	2.5–6
Lymphocytes (10^3^/µL)	2.32 ± 5.16	8.59 ± 13.59	0.577	1–4
Eosinophil (10^3^/µL)	0.35 ± 1.38	0.07 ± 0.18	0.006*	0.05–0.3
Hemoglobin (g/dL)	12 ± .2.36	12.81 ± 9.23	0.627	13.5–17.5
Hematocrit (%)	37.24 ± 7.47	37.38 ± 7.34	0.921	36–50
Platelets (10^3^/µL)	210 ± 138.88	227.40 ± 110.23	0.428	200–500
ESR (mm/hr)	47.75 ± .35.47	45.31 ± 29.39	0.801	0–29
BUN (mg/dL)	39.36 ± 22.97	21.24 ± 19.55	< 0.0001*	6–24
Creatinine (mg/dL)	2.23 ± 2.08	1.59 ± 2.20	0.129	0.74–1.35
Na (mEq/L)	139.81 ± 9.02	137.05 ± 6.06	0.028*	135–145
K (mmol/L)	4.57 ± 1.32	4.21 ± 0.66	0.017*	3.6–5.2
Ferritin (ng/mL)	2292 ± 3600	1060 ± 1742	0.006*	20–250
CRP (mg/dL)	13.11 ± 9.47	10.85 ± 11.50	0.326	0.3–1.0
PCT (ng/mL)	3.42 ± 7.00	3.44 ± 18.46	0.980	< 0.5
Lactate (mmol/L)	48.84 ± 172.95	5.42 ± 34.24	0.003*	0.5–2.2
Troponin (ng/mL)	105.81 ± 448.26	0.02 ± 0.03	0.010*	< 0.04
CK (U/L)	438.37 ± 567.66	242.80 ± 452.35	0.087	22–198
BNP (ρg/mL)	4307.76 ± 5997.9	3098.26 ± 3450.4	0.635	< 300
LDH (U/L)	606.00 ± 468.67	393.26 ± 224.11	< 0.0001*	140–280
Fibrinogen (mg/dL)	656.00 ± 153.09	538.83 ± 165.68	0.288	200–400
ALT (U/L)	107.38 ± 290.70	55.41 ± 85.80	0.048*	7–55
AST (U/L)	258.66 ± 983.07	59.26 ± 70.67	0.005*	5–40
Albumin (g/dL)	3.19 ± 0.77	3.62 ± 0.55	< 0.0001*	3.4–5.4
D-dimer (µg/mL)	5.35 ± 6.25	4.85 ± 25.40	0.936	0.05–6.5
Bilirubin (mg/dL)	0.56 ± 0.33	0.66 ± 1.09	0.625	0.3
Prothrombin (Second)	15.70 ± 2.76	14.77 ± 2.58	0.160	11–13.5
APTT (Second)	59.53 ± 48.96	36.70 ± 19.17	< 0.0001*	30–40
pH	7.34 ± 0.11	7.30 ± 0.49	0.684	7.35–4.45
PaCo2 (mm Hg)	36.28 ± 17.08	35.68 ± 12.13	0.841	38–42
FiO2_lab	75.20 ± 29.90	39.04 ± 25.53	< 0.0001*	
Bicarbonate (mEq/L)	21.18 ± 4	22.79 ± 29.90	0.140	23–30

### Predicting hospital mortality using clinical and paraclinical data

The multivariate approach showed that patients’ demographics, clinical variables, comorbidities, and biochemical markers can be used for predicting hospital mortality outcomes. SIMPLS analysis was carried using most differentiating variables (VIP > 1.0) [11] to establish the prediction model. The prediction model was developed on 172 patients in the training set and 78 patients in the validation set. Two-factor-based SIMPLS models had moderate predictability (*Q*^2^ = 0.24) with the variability of *R*^2^ = 0.37 using a total of 21 variables that contributed to the prediction models. Table 3 also shows that CAD is the most important variable associated with mortality followed by diabetes mellitus, AMS, and age > 65.

**Table 3:** Importance values (VIP) of 21 most differentiation among 108 variables used in the primary model

	Variables	VIP
1	CAD	2.1045
2	Diabetes	1.9098
3	Age > 65	1.7433
4	AMS	1.6922
5	Dementia	1.6309
6	Nursing home	1.5545
7	*Q*^2^ saturation < 88	1.5252
8	yno2	1.4903
9	Consolidation	1.4654
10	Hypertension	1.4226
11	Atrial fibrillation	1.3789
12	Alcohol	1.2563
13	Chest pain	1.1566
14	Peripheral vascular disease	1.1133
15	Prothrombin	1.0855
16	Stroke	1.0665
17	Headache	1.0412
18	Dyspnea	1.0212
19	CRP	1.0125
20	Lactate	1.0012
21	Smoking	1.0011

**Fig. 1:**
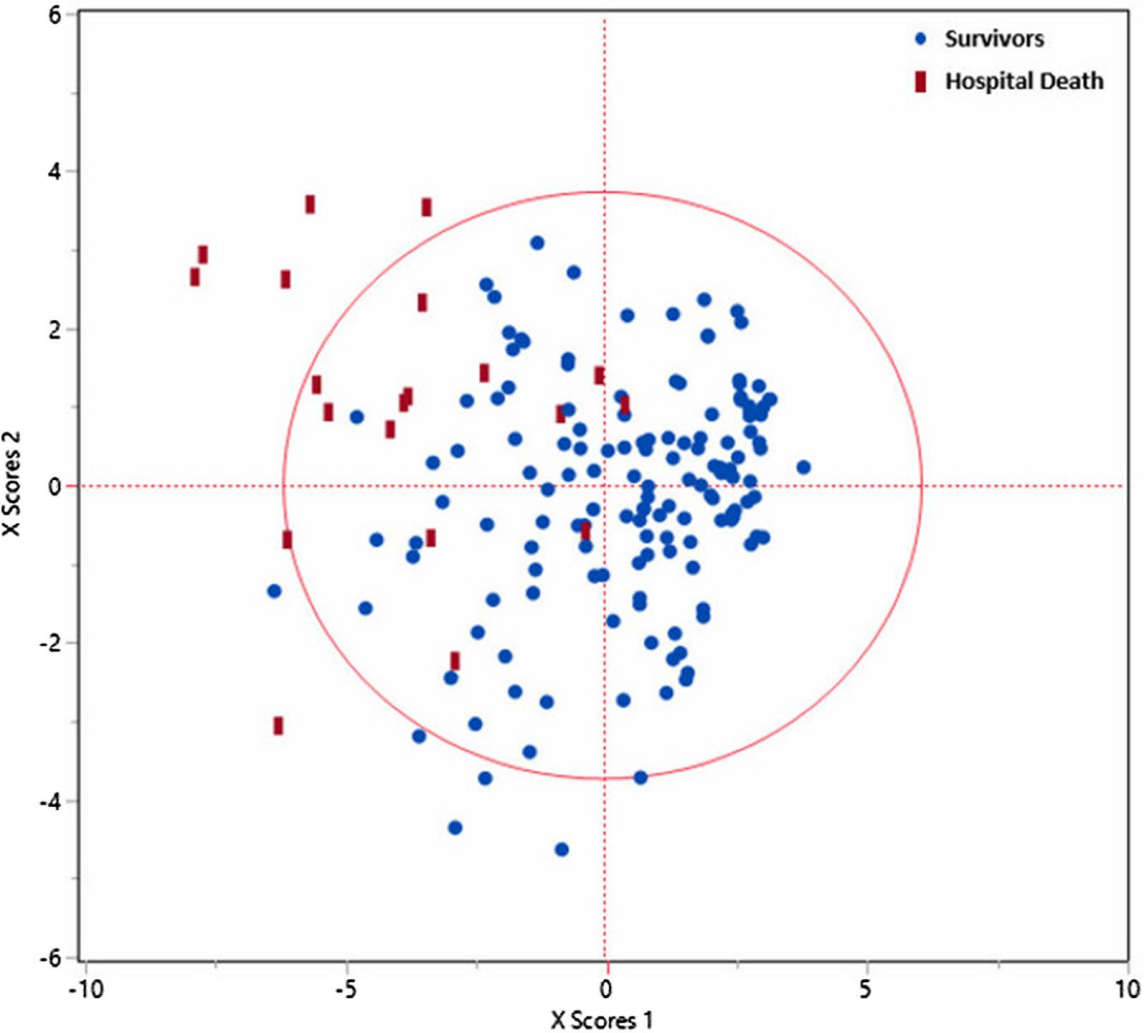
SIMPLS-based scatter plot shows a good separation between hospital mortality of patients with COVID-19 from survivors. The figure illustrates only the training set-based scatter plot

Further, the coefficient plot revealed that the age > 65, nursing home, headache, dyspnea, AMS, consolidation, O2 saturation < 88, yno2, CAD, diabetes, alcohol, hypertension, stroke, dementia, prothrombin, and CRP were positively correlated with mortality among patients with COVID-19. On the other hand, chest pain, smoking, hypertension, atrial fibrillation, and peripheral vascular disease were negatively correlated with mortality. Scatterplot using two factors is characterized by adequately discriminating between patients who died and those who survived from COVID-19 in hospital ensuring accurate prediction of clinical variables (Fig. 1).

**Fig. 2:**
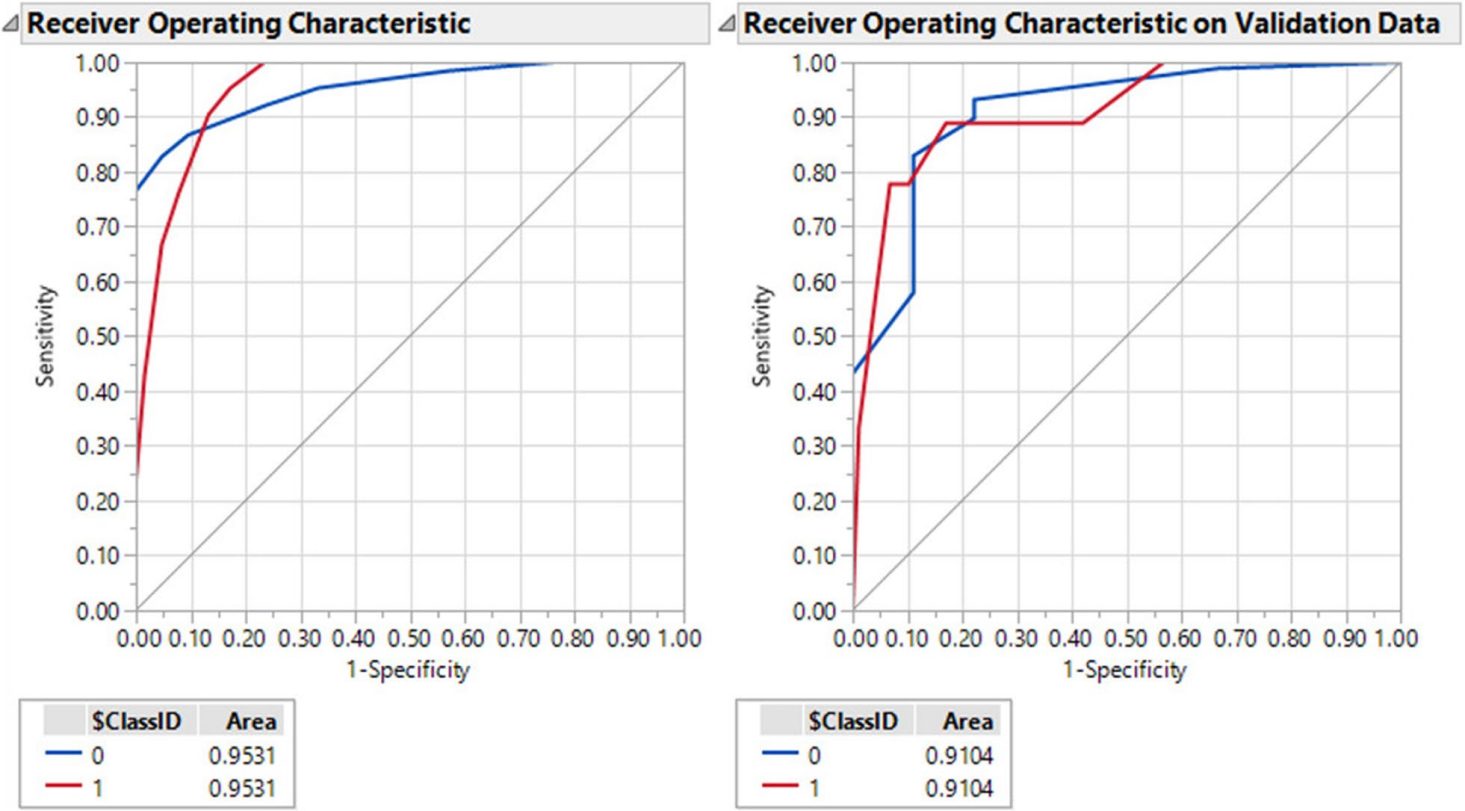
AUC for the separation of hospital mortality and survivors from COVID-19

Further multivariate correlation analysis (Table 3) showed that CAD, diabetes, hypertension, AMS, dementia, stroke, atrial fibrillation, O2 saturation < 88, yno2, nursing home, and age > 65 are correlated together and mortality. Also, O^2^ saturation < 88, lactate, dyspnea, consolidation in chest images, AMS, respiratory rate > 20 and yNO^2^ were correlated together. Age > 65, dementia, hypertension, and nursing home were closely intercorrelated. Also, the correlation analysis showed that alcohol and headache had a more negative correlation with most variables such as nursing home, diabetes, dementia, hypertension, CAD, and AMS. Only prothrombin and CRP were correlated only together, and lactate was correlated with O2 saturation < 88, yno2 and atrial fibrillation (Table 3). Predictive partition analysis verified that the above-mentioned most differentiating clinical and blood maker variables are strong predictors to partition hospital mortality and survivors according to AUC = 0.95 and AUC = 0.91 for the training and validation sets, respectively (Fig. 2). The sensitivity, specificity, and accuracy were 80%, 92%, and 90% for the training set and 75%, 90%, and 87% for the validation set, respectively.

**Fig. 3:**
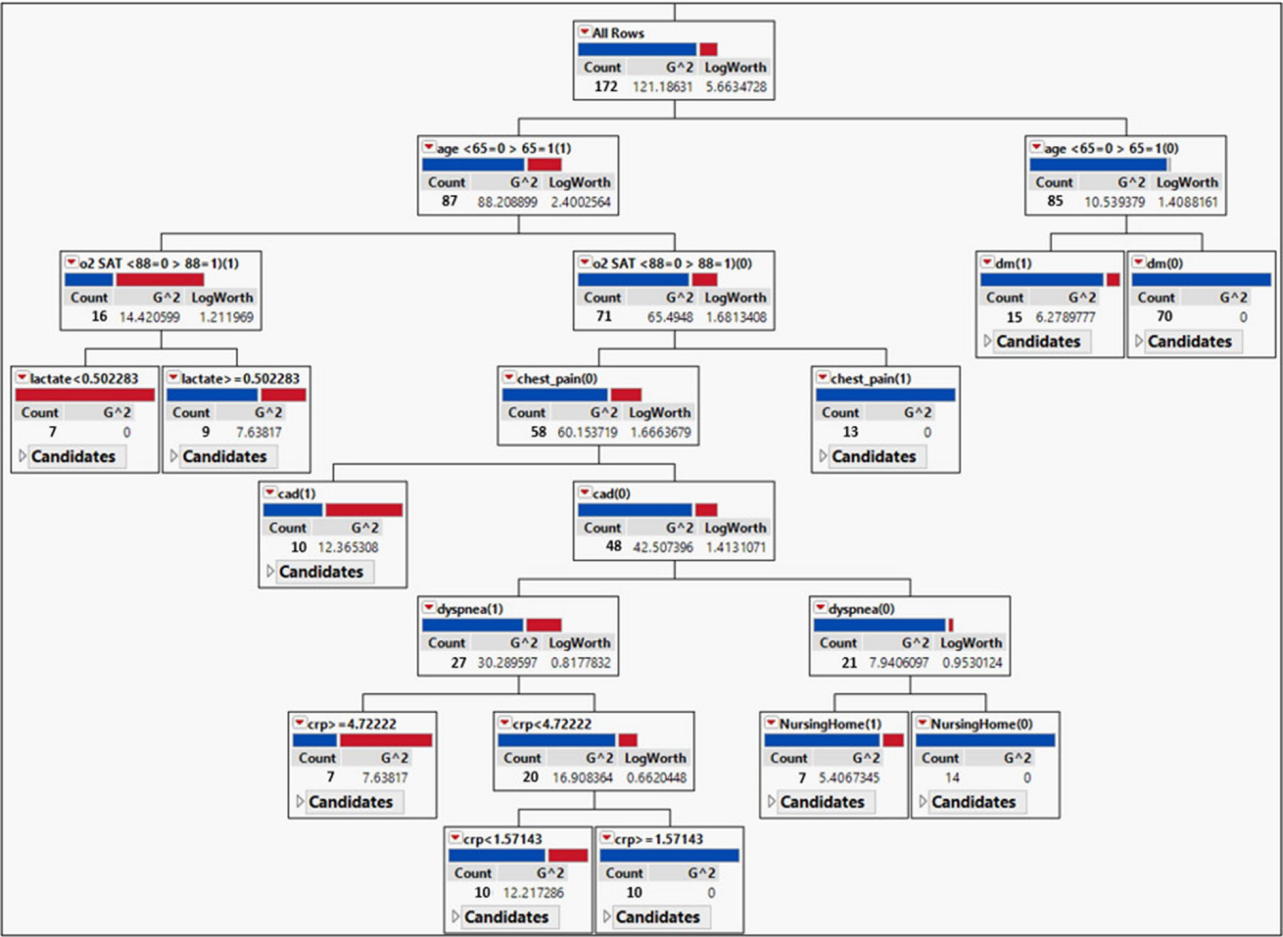
Predictive partition platform analysis shows the decision tree that predicts the hospital mortality in patients with COVID-19 from survivors. Blue square: survivors, red square: hospital mortality

Decision tree-based partition analysis revealed that age < 65 and either absence or presence of diabetes were involved to partition at least 50% of survivors. Also, age > 65, the O2 saturation condition, chest pain, and CAD had the highest portion for the partitioning of hospital death from survivors (Fig. 3).

### Identification of high-risk patients with COVID-19

**Fig. 4:**
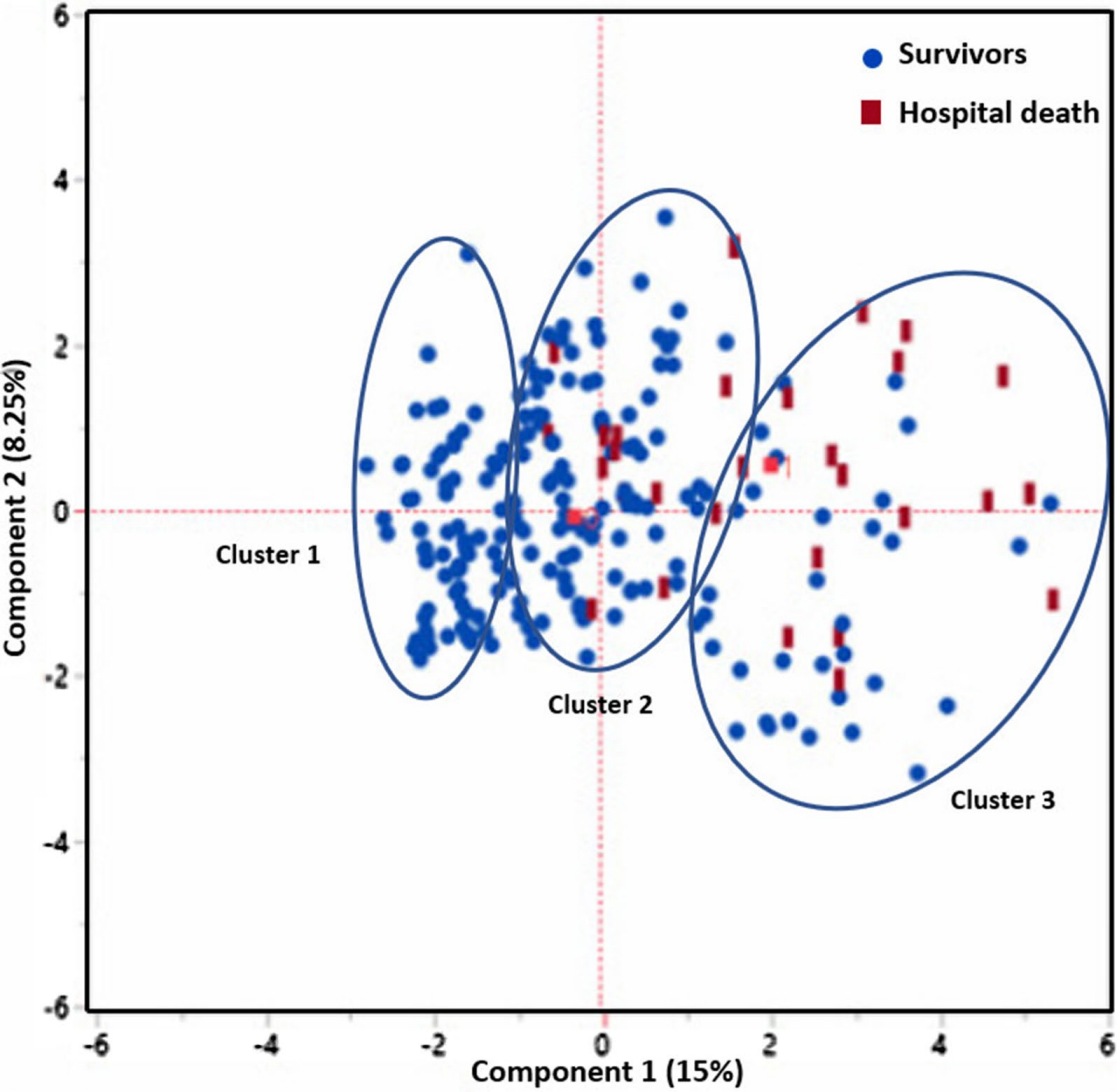
PCA plot illustrates the LCA-based clustering of patients with COVID-19. Clusters 2 and 3 are associated with a higher rate of mortality. Black circle: Survivors, red square: Hospital mortality

Further investigations using PCA and LCA showed that patients with COVID-19 can be clustered to identify the high-risk patients (Fig. 4) based on the clinical data.

LCA was performed using most differentiating clinical variables obtained by SIMPLS prediction models. LCA-based clustering revealed three main clusters among the patients with COVID-19 cohorts (survivors and non-survivors). LCA-based clustering revealed that cluster 3 and cluster 2 had a 38% and 12.5% mortality rate. Cluster 1 was with the lowest rate of mortality (0–1.3%) compared to clusters 2 and 3. All 3 clusters were well depicted through a PCA plot that can verify the clustering using two unsupervised methods. Table 4 shows that although variables had different contributions to each cluster, several variables markedly impact clustering. Hence, age < 65, lack of hypertension, lack of diabetes, alcohol consumption, and headache were highly correlated with cluster 1 and with a lower rate of mortality. On the other hand, age > 65, nursing home, AMS, stroke, atrial fibrillation, CAD, and dementia were the most important variables correlated with cluster 3; chest pain and dyspnea were the most important variables correlated with cluster 2. Also, hypertension, yno2, consolidation, O2 saturation < 88, and diabetes were variables that had a similarly high probability for clusters 2 and 3. This result showed that nursing home, dementia, O2 saturation < 88, diabetes, hypertension, age > 65 are risk factors for COVID-19 survivors in clusters 2 and 3. Table 4 shows the probability of all 18 variables for each cluster in the analysis. Multivariate correlation analysis of 19 most differentiating clinical and comorbidities predictor was obtained by SIMPLS. The correlation values > 0.2 are in red with highlighted cells (Table 5).

**Table 4:** The conditional probabilities for each cluster are shown for each response category of 20 variables in the analysis

Variable	Category	Cluster 1	Cluster 2	Cluster 3
Age > 65	No	0.8791	0.3844	0.0429
Age > 65	Yes	0.1209	0.6156	0.9571
Nursing home	No	0.9976	0.9509	0.3191
Nursing home	Yes	0.0024	0.0491	0.6809
Smoking	No	0.9157	0.9411	0.9255
Smoking	Yes	0.0843	0.0589	0.0745
Alcohol	No	0.5509	0.7871	0.9453
Alcohol	Yes	0.4491	0.2129	0.0547
Chest pain	No	0.8947	0.7753	0.996
Chest pain	Yes	0.1053	0.2247	0.004
Dyspnea	No	0.4492	0.2709	0.5011
Dyspnea	Yes	0.5508	0.7291	0.4989
Headache	No	0.8459	0.9365	0.9793
Headache	Yes	0.1541	0.0635	0.0207
AMS	No	0.9954	0.9214	0.5785
AMS	Yes	0.0046	0.0786	0.4215
Consolidation	No	0.8887	0.7795	0.727
Consolidation	Yes	0.1113	0.2205	0.273
O2 saturation < 88	No	0.9979	0.8809	0.855
O2 saturation < 88	Yes	0.0021	0.1191	0.145
yno2	No	0.8128	0.5695	0.5008
yno2	Yes	0.1872	0.4305	0.4992
CAD	No	0.9978	0.9125	0.6985
CAD	Yes	0.0022	0.0875	0.3015
Atrial fibrillation	No	0.9911	0.9019	0.7769
Atrial fibrillation	Yes	0.0089	0.0981	0.2231
Hypertension	No	0.8206	0.173	0.1622
Hypertension	Yes	0.1794	0.827	0.8378
PVD	No	0.9977	0.9447	0.8524
PVD	Yes	0.0023	0.0553	0.1476
Stroke	No	0.9892	0.976	0.6911
Stroke	Yes	0.0108	0.024	0.3089
Dementia	No	0.9981	0.9984	0.4778
Dementia	Yes	0.0019	0.0016	0.5222
Diabetes	No	0.9772	0.5565	0.4624
Diabetes	Yes	0.0228	0.4435	0.5376

No and yes values are considered as the absence and presence, respectively, for the clinical variables

**Table 5:**
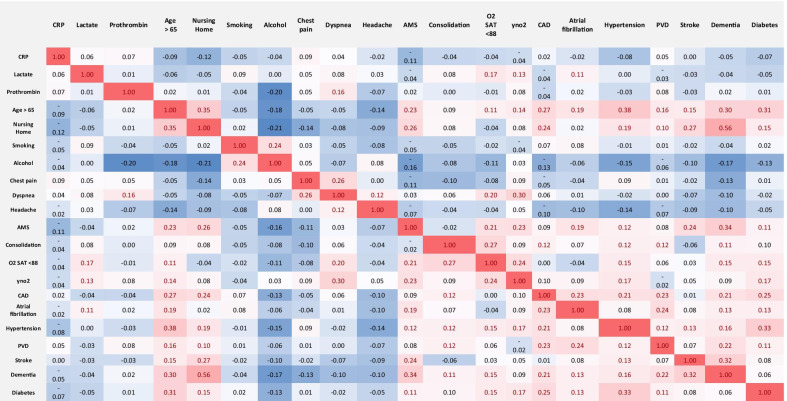
Multivariate correlation analysis of 19 most differentiating clinical and comorbidities predictor obtained by SIMPLS

The correlation values > 0.2 are in red with highlighted cells

**Fig. 5:**
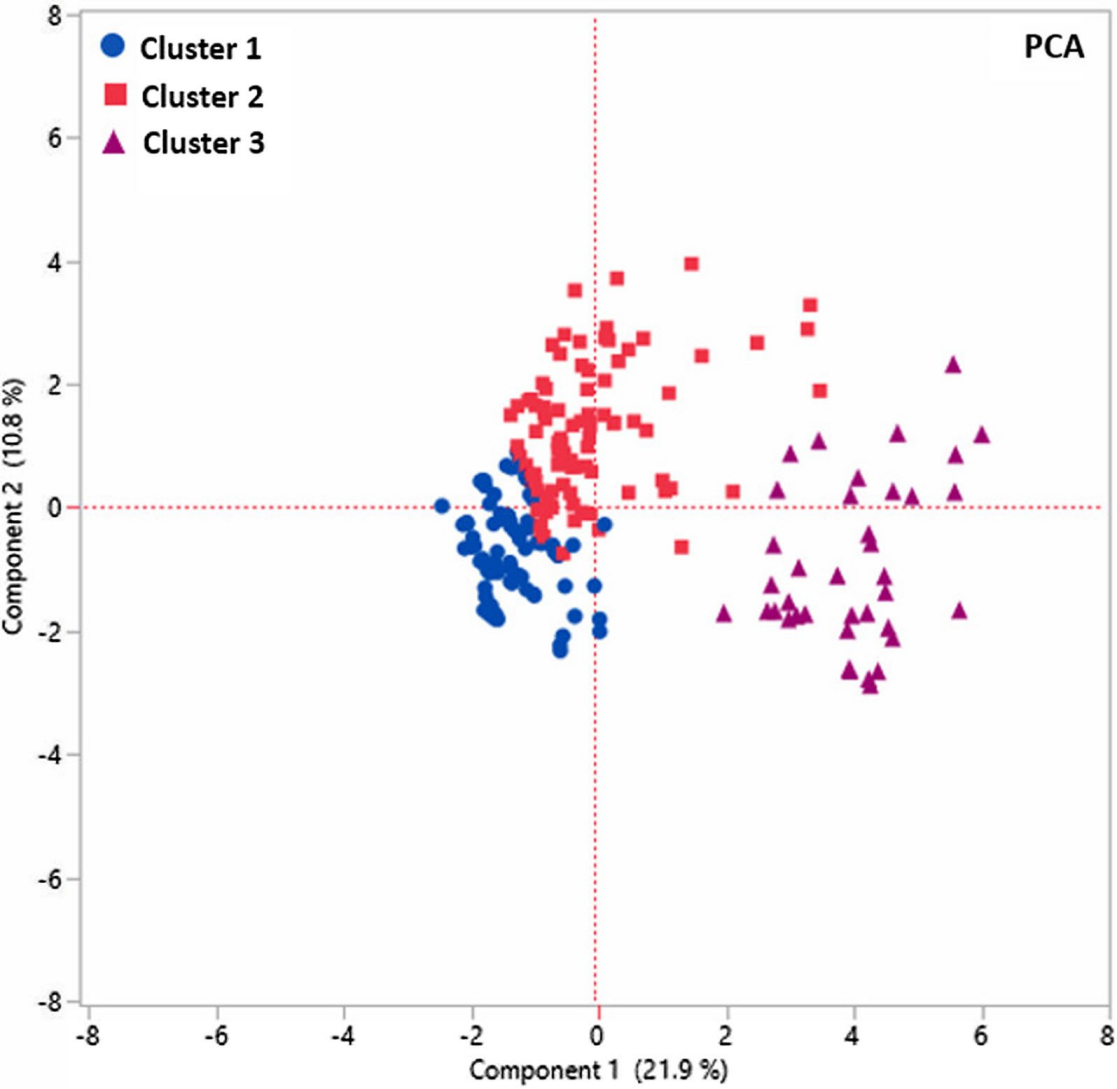
SIMPLS-based scatter plot shows a very good separation between three clusters obtained by LCA. Clusters 1 includes the patients with a lower risk of dying, and clusters 2 and 3 include patients with a higher risk of dying

Further analysis showed that three clusters are separated from each other using a very good predictive (*Q*^2^ = 0.69) with high variability (*R*^2^*Y* = 0.81) SIMPLS-based model using most differentiating variables (Fig. 5).

More investigations revealed that the prognosis of hospital mortality was poorly predicted using paraclinical data such as blood cell characteristics (i.e., numbers of leukocytes, neutrophils, lymphocytes, eosinophils, hemoglobin) and biochemical measures (i.e., BUN, creatine, sodium, CRP, procalcitonin [PCT], lactate, etc.) compared to clinical data and comorbidities.

## Discussion

In the current study, machine learning algorithms were applied to predict hospital mortality using a prediction model based on the demographic, clinical predictors, comorbidities, and biochemical markers of patients with COVID-19. The two-component SIMPLS-based prediction model had moderate predictive power *Q*^2^ = 0.24 to predict hospital mortality. The prediction model was associated with high accuracy (AUC score of 0.91–0.95) using training and validation sets of the patient cohort. The prediction model was developed based on the 18 clinical and comorbidities, and 3 paraclinical biochemical markers uncovering most differentiating predictors that some have not been recognized through conventional statistical methods. Hence, CAD showed the highest predictive importance for in-hospital death, followed by diabetes, age > 65, Altered Mental Status, dementia, and O2 saturation < 88%. Also, LCA clustering was successful to identify high- and low-risk clusters in COVID-19 survivors. The clusters were discriminated against based on the high predictive power model *Q*^2^ = 0.69. Age < 65, lack of hypertension, and lack of diabetes were highly correlated with a lower rate of mortality among survivors while residing in the nursing home, age > 65, AMS, stroke, atrial fibrillation, CAD, and dementia were risk factors for in-hospital mortality in COVID-19 survivors. Multivariate analysis demonstrated that there are some most differentiating predictors which are not included in the univariate method (Table 1) such as yno2, dyspnea, alcohol, O2 saturation, and stroke. Moreover, the multivariate analysis helped to determine the weight of the clinical predictors based on their importance in the prediction model (VIP) that is considered as the value of multivariate analysis compared to the univariate analysis. On the other hand, acute MI, CHF, O2 flow rate (lpm), Fio2, and blood pressure were significantly different between the two groups which were not selected as most differentiating predictors using SIMPLS. The combination of paraclinical data with patient demographics and comorbidities significantly improved the prediction of hospital mortality compared to when patient demographics and comorbidities or paraclinical data were independently poor predictors for the prognosis of hospital mortality. Lactate, CRP, and prothrombin were the most weighted biochemical variables that could be contributed to predicting hospital mortality.

Several other studies are published on COVID-19 mortality prediction model development. In a large cohort, Yadaw et al*.* developed a highly accurate (AUC = 0.91) ML-based mortality prediction model, using patient’s age, O2 saturation throughout their medical encounter, and type of patient encounter (inpatient versus outpatient and telehealth visits) [14]. Age and minimum O2 saturation during the encounter were the most predictive factors, which is in line with our results. Individuals aged 60 years and older represent nearly 85% of all deaths, in COVID-19 hot spots across the USA [15]. Not surprisingly, the severity of hypoxia at presentation has been extensively reported as a significant indicator of the severity of illness, specifically in acute respiratory distress syndrome, and carries strong justification to be an important predictive factor in the clinical course of COVID-19 [16, 17]. Although development and validation datasets were larger in this study, the collected data were limited to those routinely collected during hospital encounters and did not include the comprehensive list of demographics, comorbidities, biochemical tests, imaging, and omics data. Additionally, although they had large datasets, the number of dead participants was small. Knight et al*.* conducted a large prospective cohort, evaluating an 8-item scoring system (score range 0–21 points) for in-hospital mortality due to COVID-19 [18]. The variables included age, gender, number of comorbidities, respiratory rate, O2 saturation, level of consciousness, urea level, and CRP. This scoring system revealed high discrimination for mortality (derivation cohort: AUC 0.79; validation cohort: 0.77); however, some potentially relevant comorbidities such as hypertension, previous myocardial infarction, and stroke were not included in data collection. Moreover, regarding the 32.2% mortality rate and elderly patient population (median age of 73 years old), this model could function differently in younger patients and/or populations at lower risk of death.

LASSO and multivariate data analysis-based prediction models showed that higher age, coronary heart disease (CHD), percentage of lymphocytes (LYM%), procalcitonin (PCT), urea, CRP, and D-dimer (DD) could be potential risk factors for mortality of COVID. These variables could classify the COVID patients into low- and high-risk groups using a good prediction model (AUC = 0.91)[19].

Considerable heterogenicity exists among COVID-19 mortality prediction models. Unlike our results which showed paraclinical and biochemical data have limited predictive value, in the model developed by Zhao et al. (AUC 0.83), lactate dehydrogenase and procalcitonin were among the top mortality prediction factors [20], and the COVID-AID study showed that renal failure at presentation (defined by creatinine > 2 mg/dL), regardless of chronicity has a high impact on in-hospital mortality in hospitalized COVID-19 patients [21]. Recent studies have reported that prothrombin and CRP are associated with COIVD severity and mortality [22, 23]. In this study, we showed the correlation of decreased O2 and increased lactate that may indicate the higher level of the anaerobic metabolism [24] in patients with COVID-19 that are associated with mortality.

Late April 2020, a systematic review and meta-analysis showed a significantly higher rate of hypertension, diabetes, cardiovascular disease, and respiratory disease in critically ill COVID patients compared to non-critical patients [25]. Then, another systematic review and meta-analysis on risk for predicting mortality of COVID 19 patients demonstrated that dyspnea, chest tightness, hemoptysis, expectoration, and fatigue were the most significant clinical variables in association with increased risk of COVID-19 mortality. This study also showed significant increased leukocyte count and decreased lymphocyte count in non-survivors [26]. ML was successfully applied to determine COVID-19 severity by predicting the need for ICU (AUC = 0.80) and the need for mechanical ventilation (AUC = 0.82) [27]. Random forest analysis showed that PCT, DD, CRP, respiratory rate, SpO2, albumin, AST/SGOT, calcium, influenza-like symptoms, and ALT/SGPT are the most important variables to predict the need for ICU. Also, CRP, DD, PCT, SpO2, respiratory rate, creatinine, total protein, albumin, calcium, and age were the most important variables to predict the need for mechanical ventilation [27]. In a similar study, SpO2/FiO2, CRP, estimated glomerular filtration rate (eGFR), age, Charlson score, lymphocyte count, and PCT were the most important variables for the prediction COVID severity [28]. LASSO-based prediction model showed that lymphocyte percentage, lactic dehydrogenase (LDH), neutrophil count, and DD in combination with four quantitative CT findings including pneumonia percentage in the lateral basal segment of left lower lung, the volume of the whole lung with the density of -300 to -200 HU, pneumonia volume in both lungs and pneumonia volume in the right lung can be most important variables to prognosticate critical illness risk in hospitalized patients with COVID-19 pneumonia [29]. Age, PCT, CRP, LDH, DD, and lymphocytes were top mortality predictors and PCT, LDH, CRP, O2 saturation, temperature, and ferritin were important predictors for the ICU need with AUC 89% and 79%, respectively, in a cohort from New York [30].

Leon et al*.* applied the ML approach to cluster the patients with COVID into 3 groups including higher, moderate, and low rate of mortality. This study showed that the higher and lower AST, ALT, LDH, CRP, and number of neutrophils were associated with a higher and lower rate of mortality, respectively [31]. The percentages of monocytes and lymphocytes were negatively correlated with mortality [31]. Unlike our results, Leon’s study showed that age, sex, and comorbidities did not contribute to the above clustering model [31].

The strengths of our study include assessing a comprehensive list of demographic, clinical, and paraclinical variables, at all stages of hospitalization (admission, during hospital stay, and hospital discharge), development of an internally validated accurately discriminating in-hospital mortality prediction model, identification of high-risk and low-risk clusters of COVID patients whose healthcare needs are different, and enrollment of PCR-proven cases of SARS-CoV2, rather than possible COVID-19 patients. SIMPLS is considered a suitable multivariate method to investigate big and complex datasets that have a relatively small sample size and many variables [32]. External validation using an external cohort may help the results to be more practicable and achievable at any time with any cohorts. Current findings in this study may improve the precise prognostication of COVID-19 mortality, classification of low and high risk, and identification of potential risk factors.

Our study has a few limitations. First, this is a single-center retrospective study, which might impact the data quality and generalizability. Second, although we had an acceptable sample size, the subset of dead individuals was small (*n* = 31). A major reason for this concern is that the number of predictor parameters considered by ML approaches usually exceeds that for regression, even when the same set of predictors is applied, especially since multiple interaction terms are constantly examined and continuous predictors are routinely classified. Therefore, ML methodologies require “big data” to ensure their developed models have minimized overfitting and for their potential advantages (i.e., dealing with highly nonlinear relations and complex interactions) to reach fruition.

## Conclusion

In conclusion, we presented an accurate ML-based in-hospital mortality prediction model for COVID-19, which can aid in clinical decision making and resource allocation. This model needs to be externally validated in larger populations and multicenter settings.

## Data Availability

The datasets used and/or analyzed during the current study are available from the corresponding author on reasonable request.

## Abbreviations

COVID-19: Corona Virus Disease 2019
SIMPLS: Statistically Inspired Modification of Partial Least Square
LCA: Latent Class Analysis
PCA: Principal Component Analysis
CAD: Coronary Artery Disease
AMS: Altered Mental Status
ARDS: Acute Respiratory Distress Syndrome
ML: Machine Learning
AI: Artificial Intelligence
PCR: Polymerase Chain Reaction
GCS: Glascow Coma Scale
RR: Respiratory Rate
ACE: Angiotensin Converting Enzyme
COPD: Chronic Obstructive Pulmonary Disease
PE: Pulmonary Emboli
ILD: Interstitial Lung Disease
CHF: Congestive Heart Failure
AMI: Acute Myocardial Infarction
Afib: Atrial Fibrillation
CRF: Chronic Renal Failure
AUC: Area Under Curve
CRP: C-reactive Protein
PCT: Procalcitonin
DD: D-dimer
